# Identification of the high-yield monacolin K strain from *Monascus* spp. and its submerged fermentation using different medicinal plants

**DOI:** 10.1186/s40529-022-00351-y

**Published:** 2022-07-02

**Authors:** Yu-Pei Chen, Hong-Tan Wu, Ing-Er Hwang, Fang-Fang Chen, Jeng-Yuan Yao, Yiling Yin, Meng-Yun Chen, Li-Ling Liaw, Yang-Cheng Kuo

**Affiliations:** 1 Department of Public Health and Medical Technology, Xiamen Medical College, Xiamen, 361023 Fujian China; 2 Engineering Research Center of Natural Cosmeceuticals College of Fujian Province, Xiamen Medical College, Xiamen, 361023 Fujian China; 3 Institute of Respiratory Diseases Xiamen Medical College, Xiamen Medical College, Xiamen, 361023 Fujian China; 4 Bioresource Collection and Research Center, Food Industry Research and Development Institute, HsinChu, Taiwan; 5 Department of Basic Medicine, Xiamen Medical College, Xiamen, 361023 Fujian China; 6 Department of Medical Technology, Xiamen Medical College, Xiamen, 361023 Fujian China

**Keywords:** *Monascus*, Monacolin K, Antioxidant capacity, Anti-inflammatory capacity, *Glycyrrhiza uralensis*

## Abstract

**Background:**

Medical plants confer various benefits to human health and their bioconversion through microbial fermentation can increase efficacy, reduce toxicity, conserve resources and produce new chemical components. In this study, the cholesterol-lowering monacolin K genes and content produced by *Monascus* species were identified. The high-yield monacolin K strain further fermented with various medicinal plants. The antioxidant and anti-inflammatory activities, red pigment and monacolin K content, total phenolic content, and metabolites in the fermented products were analyzed.

**Results:**

Monacolin K was detected in *Monascus pilosus* (BCRC 38072), and *Monascus ruber* (BCRC 31533, 31523, 31534, 31535, and 33323). It responded to the highly homologous *mokA* and *mokE* genes encoding polyketide synthase and dehydrogenase. The high-yield monacolin K strain, *M. ruber* BCRC 31535, was used for fermentation with various medicinal plants. A positive relationship between the antioxidant capacity and total phenol content of the fermented products was observed after 60 days of fermentation, and both declined after 120 days of fermentation. By contrast, red pigment and monacolin K accumulated over time during fermentation, and the highest monacolin K content was observed in the fermentation of *Glycyrrhiza uralensis*, as confirmed by RT-qPCR. Moreover, *Monascus*-fermented medicinal plants including *Paeonia lactiflora*, *Alpinia oxyphylla*, *G. uralensis*, and rice were not cytotoxic. Only the product of *Monascus*-fermented *G. uralensis* significantly exhibited the anti-inflammatory capacity in a dose-dependent manner in lipopolysaccharide-induced Raw264.7 cells. The metabolites of *G. uralensis* with and without fermentation (60 days) were compared by LC/MS. 2,3-Dihydroxybenzoic acid, 3,4-dihydroxyphenylglycol, and 3-amino-4-hydroxybenzoate were considered to enhance the antioxidant and anti-inflammatory ability.

**Conclusions:**

Given that highly homologous monacolin K and citrinin genes can be observed in *Monascus* spp., monacolin K produced by *Monascus* species without citrinin genes can be detected through the complementary methods of PCR and HPLC. In addition, the optimal fermentation time was important to the acquisition of antioxidants, red pigment and monacolin K. These bioactive substances were significantly affected by medicinal plants over fermentation time. Consequently, *Monascus*-fermented *G. uralensis* had a broad spectrum of biological activities.

**Supplementary Information:**

The online version contains supplementary material available at 10.1186/s40529-022-00351-y.

## Background

*Monascus* species are traditionally used in fermenting red yeast rice or red koji and widely used in the food industry in Asian countries (Farkouh and Baumgartel 2019). According to the National Center for Biotechnology Information, 19 typical *Monascus* species have been identified. *Monascus purpureus*, *Monasucs pilosus*, and *Monascus ruber* are frequently used in research. Many pigments, such as rubropunctatin, monascorubrin, rubropunctamine, monascorubramine, monascin, and ankaflavin, are detected in *Monascus* species (Liu et al. 2018). They are important food colorant additives. Moreover, the pharmacological efficacy of *Monascus* species has attracted considerable interest for the improvement of health in lipid metabolism. Monacolin K, also known as lovastatin, has a cholesterol-lowering effect and can be found in *Monascus* species (Yanli and Xiang 2020). In addition to lowering lipid level, monacolin K prevents many diseases, such as colon, gastric, breast, lung, and thyroid cancer; acute myeloid leukemia; Parkinson’s disease; schizophrenia; depression; and type I neurofibromatosis (Chen et al. 2015; Hong et al. 2008; Lin et al. 2015; Xiong et al. 2019; Zhang et al. 2019b). Therefore, monacolin K production through the addition of linoleic acid, non-ionic surfactant, and glutamic acid has been extensively explored (Huang et al. 2018; Yang et al. 2021; Zhang et al. 2019a). A monacolin K biosynthetic gene cluster containing nine genes (*mokA–mokI*) is homologous to the lovastatin gene cluster from *Aspergillus terreus* (Chen et al. 2008b). Monacolin K production by *Monascus pilosus* can be enhanced by *mokH* overexpression and up-regulation of monacolin K biosynthetic genes (Chen et al. 2010b).

Probiotic-fermented food offers health benefits through the bioconversion of dairy products (Lee et al. 2021). Rice or different grains are generally used as substrates for *Monascus* fermentation to improve secondary metabolites. Extracts from *Monascus*-fermented soybean have antioxidant capacities and inhibitory activities against enzymes related to skin aging (Jin and Pyo 2017). The antioxidant activities of scavenging free radicals have been observed in *Monascus*-fermented coix seed (Zeng et al. 2021). Fish bone as an antioxidant-active peptide source can be fermented by *M. purpureus*, which increases 2,2-diphenyl-1-picrylhydrazyl (DPPH) and 2, 2′-azino-bis (3-ethylbenzothiazoline-6-sulfonic acid)(ABTS) radical scavenging ability (Chen et al. 2021). Additionally, *Monascus*-submerged fermentation using agro-industrial residues, such as rice flour and molasses, is a promising method for red pigment production (Da Silva et al. 2021). Different cereal substrates through the solid-state fermentation of *M. purpureus* have been developed for pigment production (Srianta et al. 2016). Besides, millet is a good candidate for monacolin K production using *Monascus* species (Maric et al. 2019; Zhang et al. 2018a).

Medicinal plants offer various benefits to human health. Medicinal plant bioconversion through fermentation can enhance medicinal effects by increasing efficacy, reducing toxicity, conserving resources, and producing new chemical components (Li et al. 2020a). Microbes used in the fermentation of medicinal plants include *Bacillus*, lactic acid bacteria, yeast, and filamentous fungi. By contrast, natural fermentation without additional microbes is a common method but less effective and specific than other known methods. In this study, a high-yield monacolin K strain from 18 *Monascus* spp. was screened and identified. Subsequently, medicinal plants for treating diarrhea, dementia, pain, cold, inflammation, and immune disorders were used as substrates for *Monascus* fermentation. The fermentation products were used in analyzing antioxidant and anti-inflammatory activities, red pigment, monacolin K, and total phenolic content. Metabolites from the fermentation product with the best efficacy were further analyzed through LC/MS.

## Materials and methods

### *Monascus* spp., medicinal plants, and growth conditions

*Monascus* spp. listed in Table 1 were used in this study. To detect a high-yield monacolin K strain, the medium with 7% glycerol, 3% glucose, 3% monosodium glutamate, 1.2% polypetone, 0.2% NaNO_3_, and 0.1% MgSO_4_ ·7H_2_O was performed for *Monascus* spp. incubation. Mycelia were harvested after 14 days of cultivation at 25 ℃ for DNA manipulation. Medicinal plants including *Angelica pubescens*, *Pogostemon cablin*, *Paeonia lactiflora*, *Alpinia oxyphylla*, *Melaleuca leucadendron*, *Lavandula angustifolia*, *Osmanthus fragrans*, *Glycyrrhiza uralensis*, *Phellodendron chinense*, and rice as the control were utilized for the fermentation materials (Beijing Tongrentang Traditional Chinese Medicine Co., Ltd, Chengdu, China). Two grams of medicinal plants as the sole substrate ground into a powder with 50 mL reverse osmosis water were sterilized at 121 ℃ for 30 min. Spores of *Monascus* spp. harvested from potato dextrose agar plate were added into the medicinal plant liquids for submerged batch-fermentation. *Monascus* spp. with medicinal plants were stood for 60 and 120 days at 25 ℃. After 60 and 120 days of submerged fermentation, the culture was centrifugated and filtered, and the suspension was freeze-dried. The freeze-dried powders were carried out for the bioactive assays.

**Table 1:** Strains used in this study and their monacolin K characteristic

Strain	Species^a^	Polyketide synthase (*mokA*)^b^	Dehydrogenase (*mokE*)	Monacolin K production (mg/g mycelia)
1. BCRC 31,502 (ATCC 16363)	*Monascus pilosus*, Type	+	+	—
2. BCRC 38072	*Monascus pilosus*	+	+	0.151 ± 0.111
3. BCRC 31533 (ATCC 16246)	*Monascus ruber*, Type	+	+	0.156 ± 0.031
4. BCRC 31523 (ATCC 16378)	*Monascus ruber*	+	+	0.019 ± 0.032
5. BCRC 31534 (ATCC 16366)	*Monascus ruber*	+	+	0.180 ± 0.137
6. BCRC 31535 (ATCC 18199)	*Monascus ruber*	+	+	0.924 ± 0.262
7. BCRC 33314 (ATCC 16371)	*Monascus ruber*	+	+	—
8. BCRC 33323 (ATCC 18199)	*Monascus ruber*	+	+	0.282 ± 0.068
9. BCRC 31542 (ATCC 16365)	*Monascus purpureus*, Type	—	—	—
10. BCRC 31541 (ATCC 16379)	*Monascus purpureus*	—	—	—
11. BCRC 33325 (IFO 30873)	*Monascus purpureus*	—	—	—
12. BCRC 31615 (DSM 1379)	*Monascus purpureus*	—	—	—
13. BCRC 31506 (CBS 302.78)	*Monascus kaoliang*, Type	—	—	—
14. BCRC 33446 (ATCC 200613)	*Monascus sanguineus*, Type	—	—	—
15. BCRC 33309 (ATCC 16966)	*Monascus barkeri*	+	+	—
16. BCRC 33310 (IMI 282587)	*Monascus floridanus*, Type	—	—	—
17. BCRC 33640 (ATCC 204397)	*Monascus lunisporas*, Type	—	—	—
18. BCRC 33641 (ATCC 200612)	*Monascus pallens*, Type	—	—	—
19. BCRC 32670 (ATCC 20542)	*Aspergillus terreus*	+	+	0.228 ± 0.199

^a^“Type” indicates type strain

^b^+, positive; −, negative

### DNA manipulation

Approximately 0.5 g *Monascus* mycelia were ground using a mortar and pestle by liquid nitrogen. DNA was extracted by phenol and chloroform, and precipitated by isopropanol. DNA was finally dissolved in TE buffer. PCR was implemented according to the condition by Chen et al. (2008a). The primer sets of *mokA* and *mokE* genes involved in the monacolin K biosynthesis were *mokA*-F, ATCATTCTTTCCNCGCTCCA, *mokA*-R, CGGGCTATTGTCGGCCATAG; *mokE*-F, GTGGTGGACTCGACGTTGGT, and *mokE*-R, TTCTCGCAGTACACGGTCAC. PCR was performed by an ABI 2700 PCR (Applied Biosystems, Thermo Fisher Scientific, Waltham, MA) and the reaction condition was as follows, 96 ℃ for 5 min by 1 cycle, 96 ℃ for 1 min, 50 ℃ for 1 min, and 72 ℃ for 1 or 2 min by 30 cycles, and 72 ℃ for 10 min with a final extension. The PCR products were recovered from agarose for DNA sequencing.

### Monacolin K detection by HPLC

The culture of *Monascus* spp. fermentation was filtered by a 0.2 mm filter. Monacolin K was determined by a high-performance liquid chromatography (HPLC) (Shimadzu, Kyoto, Japan) fitted with a reverse-phase C18 column (InsertSustain, 5 μm, 4.6 × 150 mm)(GL science Inc., Tokyo, Japan). The HPLC reaction was followed by 0.1% phosphorus acid in water with 35% and methanol with 65% at a flow rate of 1 mL/min. Monacolin K was scanned by a UV spectroscopy from 210 to 400 nm.

### Antioxidant analysis of fermentation product

The radical scavenging assay of DPPH and ABTS was used to estimate the antioxidant capacity of the fermentation product. The fermentation product of *Monascus* spp., 3,4-dihydroxyphenylglycol, and 3-amino-4-hydroxybenzoate were respectively mixed with 0.2 mM DPPH solution for 30 min and the mixture was measured by an enzyme-linked immunosorbent assay (ELISA) reader (Molecular Devices, Sunnyvale, CA) at OD_517_. The ABTS radical scavenging capacity was evaluated by the T-AOC Assay Kit (Beyotime Biotechnology, Shanghai, China). The reaction was detected by the absorbance of OD_405_. The radical scavenging activity of DPPH and ABTS (%) was calculated as follows, the radical scavenging activity (%) = ([OD_517 or 405_ of control-OD_517 or 405_ of fermentation product]/OD_517 or 405_ of control ) × 100. The sterile H_2_O was utilized as the control.

### Cell viability of fermentation product

The Raw264.7 macrophages were carried out to implement the 3-(4,5-cimethylthiazol-2-yl)-2,5-diphenyl tetrazolium bromide (MTT) assay. The cell in a 96-well plate was incubated at 37 °C for 24 h with 5% CO_2_ incubation. The fermentation products, 3,4-dihydroxyphenylglycol, and 3-amino-4-hydroxybenzoate were respectively added into the cells while the sterile H_2_O was utilized as the control. After 24 h incubation, the supernatant medium was removed and the fresh medium containing 10 µL MTT (5 mg/mL) was added into the 96-well plate of the cells for 4 h incubation. Then, the supernatant medium was discarded, and dimethyl sulfoxide was added to the 96-well plate. The mixture was detected by the absorbance of OD_570_.

### Anti-inflammatory analysis of fermentation product

The Raw264.7 macrophages were incubated at 37 °C for 24 h and 5% CO_2_ incubation in a 96-well plate. The fermentation products, 3,4-dihydroxyphenylglycol, and 3-amino-4-hydroxybenzoate were respectively added into the 96-well plate of the cells for 1 h. To stimulate the inflammation response, 1 µg/mL lipopolysaccharide was added into the cells. After 24 h incubation, the nitrite of the medium was determined by the nitrite detection kit (Beyotime Biotechnology) using the Griess reagent. The mixture was detected by the absorbance of OD_540_. The NO decreasing rate was evaluated as follows, NO decreasing rate (%) = ([OD_540_ of control-OD_540_ of fermentation products, 3,4-dihydroxyphenylglycol, and 3-amino-4-hydroxybenzoate]/OD_540_ absorbance of control) × 100. The sterile H_2_O was utilized as the control.

### Red pigment analysis of fermentation product

The freeze-dried products of *Monascus* spp. fermentation was dissolved by 1 mL 70% ethanol and centrifugated at 15,000×*g* (Hettich, Mikro 220R, Germany) for 10 min. The supernatant was obtained and detected by the absorbance of OD_505_. The red pigment was calculated as follows, red pigment (U/g) = OD_505_ of fermentation product × (1/gram) × (1/volume) × dilution factor.

### Monacolin K biosynthetic gene expression by RT-qPCR

Approximately 0.5 g *Monascus* mycelia were ground and performed by TRIzol reagent (Thermo Fisher Scientific). Total RNA was extracted by chloroform and treated with RNase-free DNase. Finally, total RNA was preserved in DEPC H_2_O. HiFi-MMLV cDNA kit (Beijing ComWin Biotech Co., Ltd., Beijing, China) with oligo (dT) and random hexamers was used to carry out the first-strand cDNA. The qPCR in a final volume of 25 µL was implemented using UltraSYBR Mixture kit (Beijing ComWin Biotech) by the Roche LightCycler® 480 System (Roche Group, Switzerland). The primer sets of monacolin K biosynthetic genes were utilized as follows, q*mokA*-F, CTGGTGCAGACACAGTACGACAT, q*mokA*-R, GGAACCATCGCCGACAAAT; q*mokB*-F, GGGACCCTGAGTTTCGAACA, q*mokB*-R, GCACTTTTTCACCCCGTTGA; q*mokC*-F, GAGGCCAGCGCGACAAT, q*mokC*-R, GTGACAGTGCGTGTCACCAAA; q*mokD*-F, CCGCTTTACGGGAAGACTTT, q*mokD*-R, GAACCCTCGAACCAGGTGTA; q*mokE*-F, GCGACGATTGTGATGCAGAT, q*mokE*-R, ATCTTCTGCGCCGTGCTTT; q*mokF*-F, AACGGAGAAGCAGATGAACCA. q*mokF*-R, TCCCACCAAGCCCAAAACT; q*mokG*-F, CGTCCGGAAGGTCCTGAAG, q*mokG*-R, TGAACCCCCCCATACTACCA; q*mokH*-F, GGAGTGGCCAAAACAGGAAA, q*mokH*-R, TGCGGGTGTTGGATTGTTG; q*mokI*-F, TGCTGGGAGGTGCTTTTACC, and q*mokI*-R, AATGTGGATGGCGAGAAGGA. 18 S rRNA was used as the internal control and the primer set was mpF18S rRNA, TCTCGTAATCGGAATGAGAACGA, and mpR18S rRNA, TACGCTATTGGAGCTGGAATTACC. The three replicates of gene expression were done and calculated by the 2^−ΔΔCT^ method.

### Analysis of total phenol and metabolites from the fermentation product

Total phenol of fermentation product was measured by the Folin-Ciocalteu method. The fermentation product was added into Folin-Ciocalteu reagent for 5 min reaction, and then 10% sodium carbonate solution was used for color development in darkness. The gallic acid of different concentrations as a positive control was utilized for the calculation of the standard curve. The mixture was centrifugated at 15,000×*g* for 10 min and the supernatant was measured by the absorbance of OD_760_. Thermo Vanquish system equipped with an ACQUITY UPLC® HSS T3 (150 × 2.1 mm, 1.8 μm, Waters Corporation, Milford, MA) column was accomplished for the analysis of fermentation product. The UPLC reaction was followed by 0.1% formic acid in acetonitrile (A) and 5 mM ammonium formate in acetonitrile (B) at a flow rate of 0.25 mL/min. The gradient of solvent A/B (v/v) was set as follows, 2% A/B from 0 to 1 min; 2-50% A/B from 1 to 9 min; 50–98% A/B from 9 to 12 min; 98% A/B from 12 to 13.5 min; 98–2% A/B from 13.5 to 14 min; 2% A from 14 to 20 min for positive model (2% B from 14 to 17 min for negative model). The ESI-MS was implemented by the Q Exactive Plus mass spectrometer (Thermo Fisher Scientific) with the spray voltage of 3.5 kV and − 2.5 kV in positive and negative modes, respectively. Data-dependent acquisition MS/MS was carried out with the HCD scan and the normalized collision energy was 30 eV. Unnecessary information in MS/MS spectra was removed by dynamic exclusion. The metabolites were screened by the accurate molecular weight (molecular weight error < 30 ppm) and identified by Metlin (http://metlin.scripps.edu), Xue et al. (2020) MoNA (https://mona.fiehnlab.ucdavis.edu//) and the database (built by Azenta Life Sciences, Suzhou, China) according to MS/MS fragment mode.

### Statistical analysis

Duncan’s multiple range test, Pearson correlation, and repeated-measures analysis of variances (ANOVAs) were performed by IBM SPSS Statistics v20 software package (SPSS Inc. Chicago, USA) at a confidence level of 95%. The mean and standard deviation of three replicates were shown.

## Results

### Identification of monacolin K biosynthetic genes and content from *Monascus* spp.

**Fig. 1:**
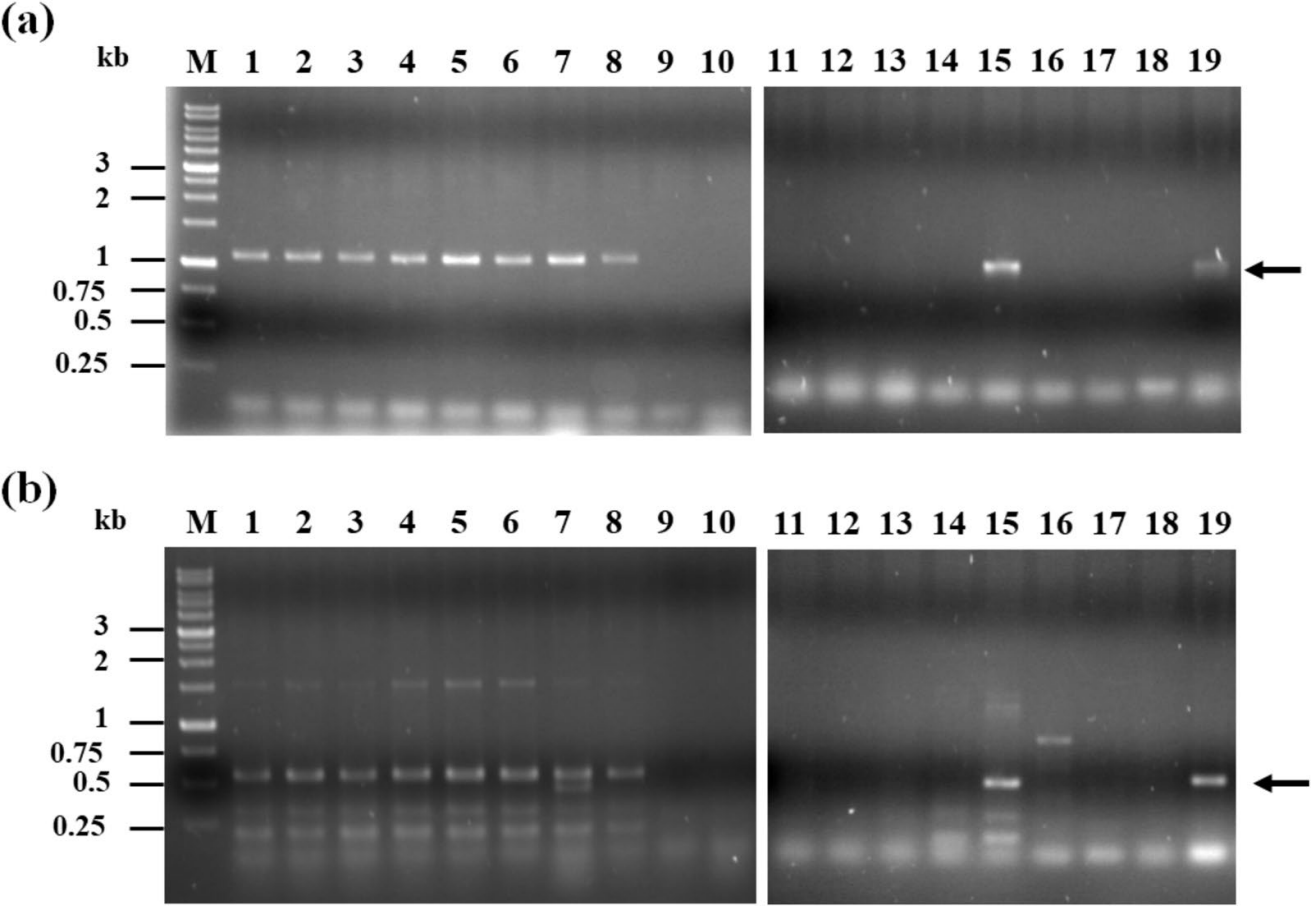
PCR analyses of monacolin K-related genes. The partial *mokA* (**a**) and *mokE* (**b**) genes were detected. Lanes 1–19 revealed *M. pilosus* BCRC 31502, and 38072; *M. ruber* BCRC 31533, 31523, 31534, 31535, 33314, and 33323; *M. purpureus* BCRC 31542, 31541, 33325, and 31615; *M. kaoliang* BCRC 31506; *M. sanguineus* BCRC 33446; *M. barkeri* BCRC 33309; *M. floridanus* BCRC 33310; *M. lunisporas* BCRC 33640; *M. pallens* BCRC 33641; *A. terreus* BCRC 32670. The arrows indicated the expected DNA bands by PCR

*mokA* and *mokE* genes encoding polyketide synthase and dehydrogenase involved in monacolin K biosynthesis from 18 *Monascus* spp. and one *Aspergillus terreus* were amplified through PCR. The expected bands of approximately 1 and 0.5 kb were detected (Fig. 1). The partial *mokA* and *mokE* genes can be obtained from *M. pilosus* (BCRC 31502 and 38072), *M. ruber* (BCRC 31533, 31523, 31534, 31535, 33314, and 33323), and *Monascus barkeri* (BCRC 33309). A similar result was obtained in *A. terreus* BCRC 32670, which can produce lovastatin and has *lov* genes. The amplified PCR products were further sequenced. Highly homologous DNA sequences were obtained in the partial *mokA* and *mokE* genes (Additional file 1: Fig. S1). However, *lovB* and *lovC* genes from *A. terreus* showed some differences from those of *Monascus* spp. *mlcA* and *mlcG* derived from the compactin biosynthetic gene cluster of *Penicillium citrinum*, which had a gene cluster and cholesterol-inhibiting efficacy similar to monacolin K, were different from *mokA* and *mokE* (Abe et al. 2002).

*Monascus* spp. and *A. terreus* BCRC 32670 were incubated for 14 days, and monacolin K content was determined (Table 1). Monacolin K was detected in *M. pilosus* (BCRC 38072), *M. ruber* (BCRC 31533, 31523, 31534, 31535, and 33323), and *A. terreus* BCRC 32670 in response to the PCR results. *M. ruber* BCRC 31535 had the highest monacolin K content. Hence, it was used in submerged fermentation with different medicinal plants.

### Antioxidant analysis of *M. ruber* BCRC 31535-fermented product

**Fig. 2:**
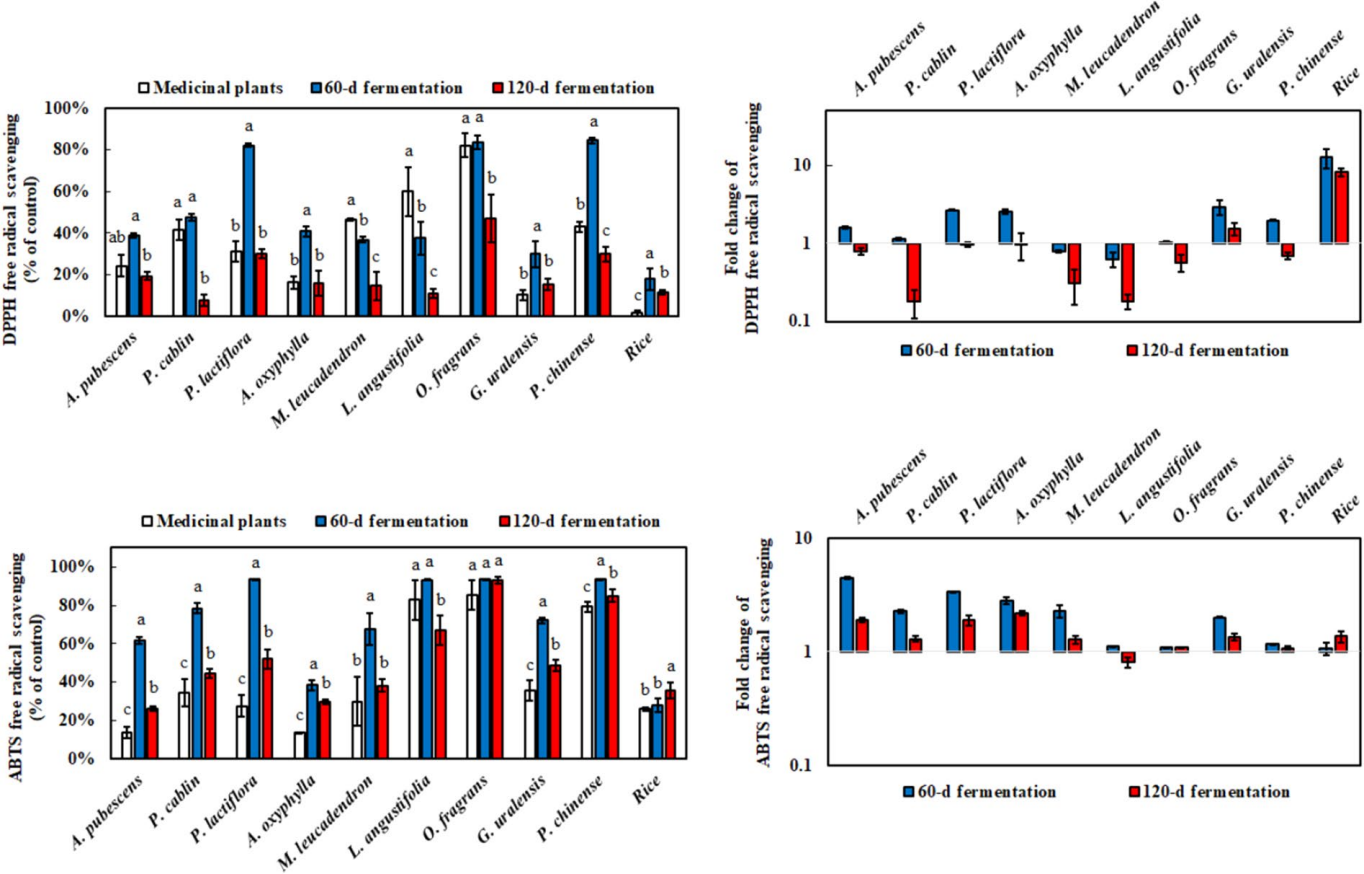
Antioxidant capacity determined using DPPH and ABTS scavenging methods with or without the fermentation of medicinal plants at 0.5 and 0.0625 mg/mL by *M. ruber* BCRC 31,535. The ratio of 60 and 120 days of fermentation to medical plants was depicted on the right side of the figure. Results are mean ± S.D. (n = 3)

Nine medicinal plants and one rice were used in the submerged fermentation of *M. ruber* BCRC 31535 for antioxidation, anti-inflammation, and metabolite analysis. The extracts of nine medicinal plants without *M. ruber* BCRC 31535 fermentation exhibited antioxidant capacity, especially in *L. angustifolia* and *O. fragrans*, which scavenged over 50% of DPPH free radicals at 0.5 mg/mL (Fig. 2). However, rare antioxidation was detected in rice. On the other hand, the effect of these products at 0.5 mg/mL on the antioxidant activity of ABTS free radical were not compared because all of them had ABTS scavenging rates of over 90%. Nevertheless, the ABTS scavenging capacity of the medicinal plants had a similar antioxidant profile to DPPH result at 0.0625 mg/mL. After the 60-day fermentation of *M. ruber* BCRC 31535, the DPPH scavenging ability of the fermentation product was elevated, except in *M. leucadendron*, and *L. angustifolia*. In addition, the order of the DPPH scavenging capacity by fold change was rice > *G. uralensis* > *P. lactiflora*, whereas that of ABTS was *A. pubescens* > *P. lactiflora* > *A. oxyphylla* in the 60-day fermentation. However, the ABTS and DPPH scavenging capacities significantly declined after 120 days submerged fermentation.

### Anti-inflammatory analysis of *M. ruber* BCRC 31535-fermented product

**Fig. 3:**
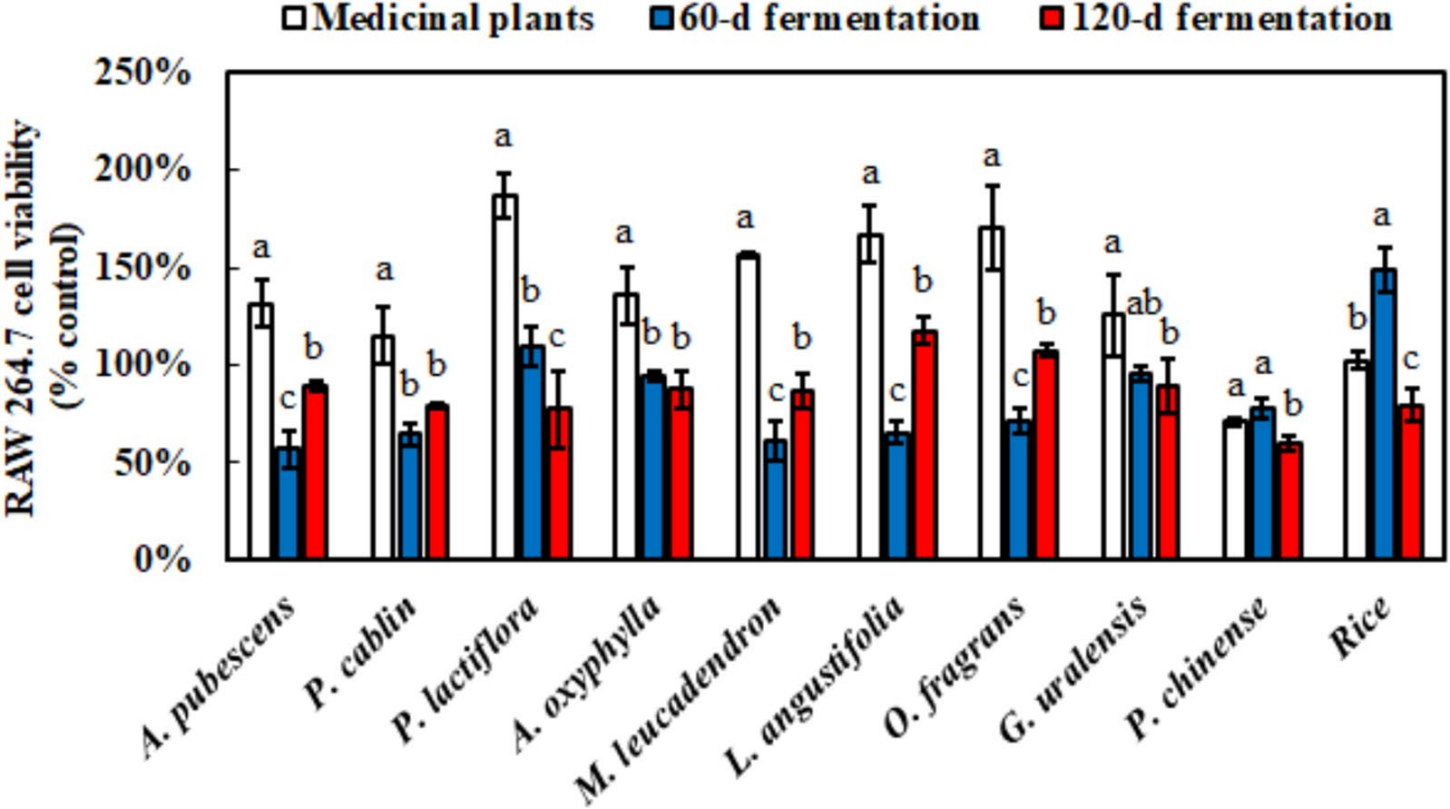
Raw264.7 cell viability using MTT method with or without fermentation of medicinal plants at 0.5 mg/mL by *M. ruber* BCRC 31535. Results are mean ± S.D. (n = 3)

**Fig. 4:**
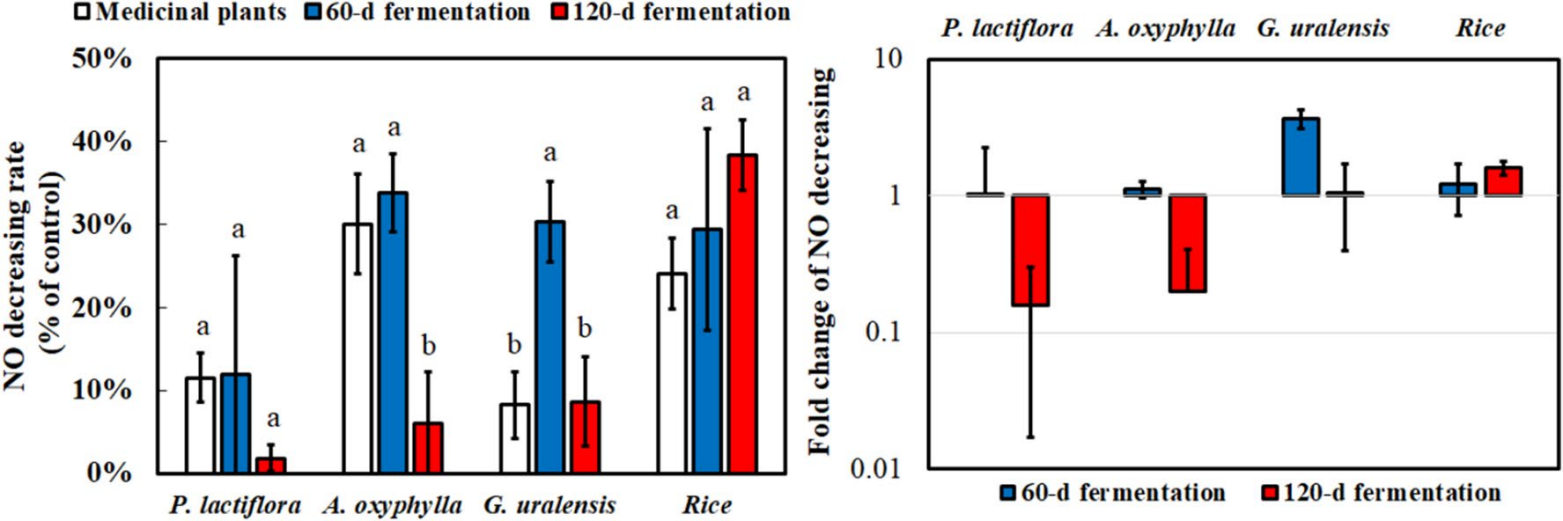
Anti-inflammatory capacity using LPS-stimulated Raw264.7 cell with or without fermentation of medicinal plants at 0.5 mg/mL by *M. ruber* BCRC 31535. The ratio of 60 and 120 days of fermentation to medical plants was depicted on the right side of the figure. Results are mean ± S.D. (n = 3)

To analyze the anti-inflammatory capacity of *M. ruber* BCRC 31535-fermented product, the cell viability of the fermentation product was explored (Fig. 3). Most medicinal plants without fermentation were harmless to Raw264.7 cells, and cell viability below 80% was observed only in *P. chinense*. However, the cell viability of the fermentation products significantly decreased, and *M. ruber* BCRC 31535-fermented *P. lactiflora*, *A. oxyphylla*, *G. uralensis*, and rice had survival rates of over 90% after 60 days of fermentation. Thus, their anti-inflammatory effects were further investigated using their fermentation products (Fig. 4). *A. oxyphylla* without fermentation showed the best anti-inflammatory effect. After the fermentation of *M. ruber* BCRC 31535, the fermented *G. uralensis* significantly improved anti-inflammatory capacity. The trend of anti-inflammatory effect of the fermentation products was similar to that of their antioxidant ability. After 120 days of submerged fermentation, anti-inflammation obviously decreased.

### Red pigment and monacolin K analysis of *M. ruber* BCRC 31535-fermented product

**Fig. 5:**
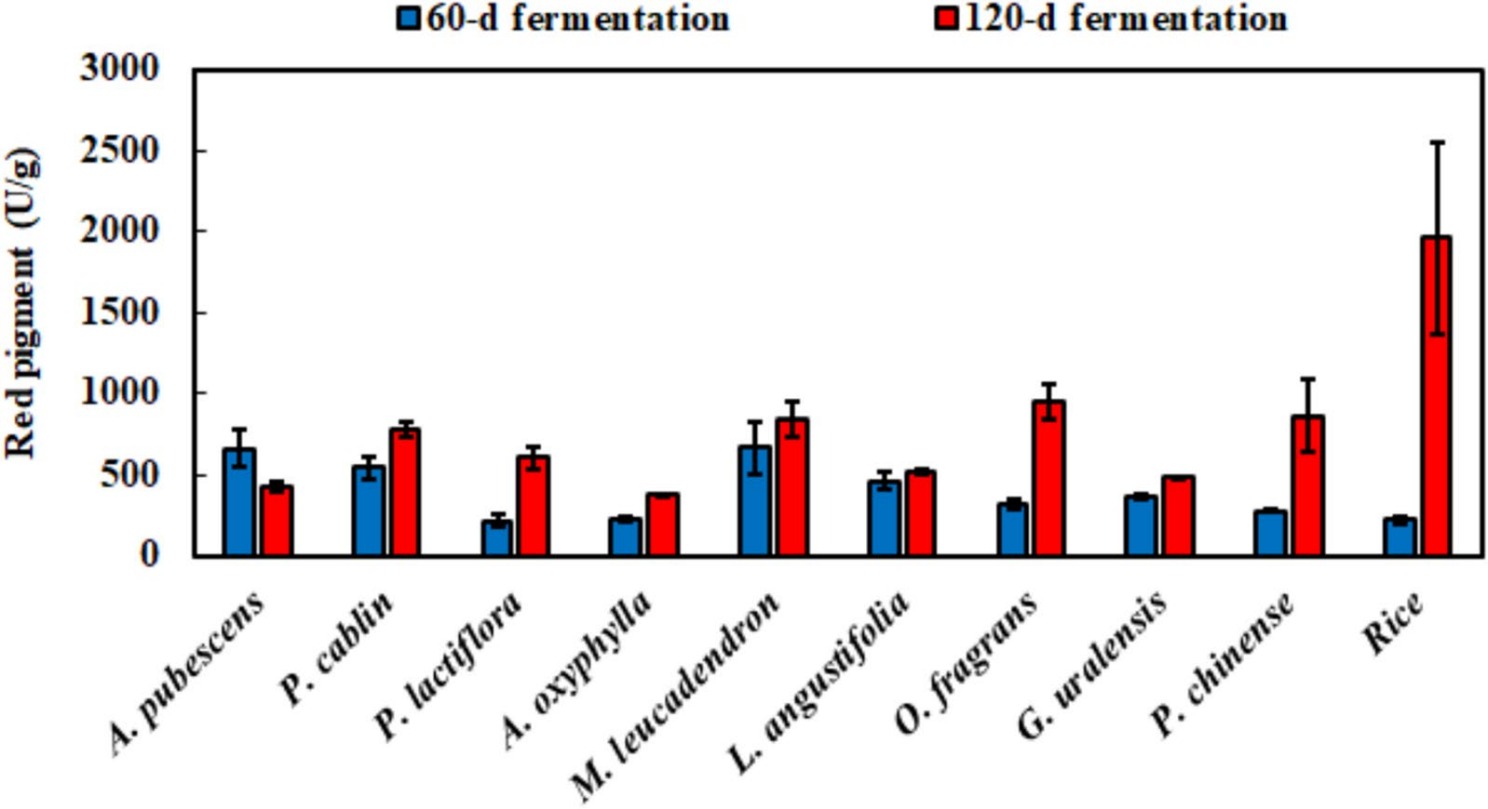
Red pigment with the fermentation of medicinal plants by *M. ruber* BCRC 31,535 and detected by the absorbance of OD_505_. Results are mean ± S.D. (n = 3)

Red pigment and monacolin K derived through polyketide synthesis can be produced by *M. ruber* BCRC 31,535 and these of fermentation product were further determined. The red pigment of the fermentation product increased with fermentation time, except in *A. pubescens* (Fig. 5). The red pigment of the 120-day fermentation product from rice was 8.9-fold that of the 60-day fermentation product.

**Fig. 6:**
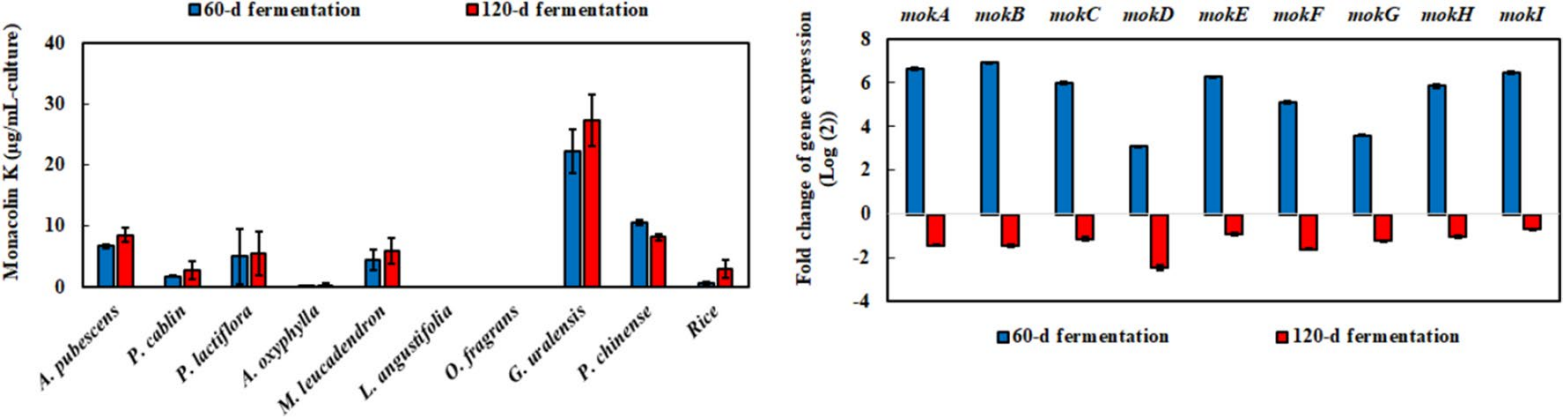
Monacolin K with the fermentation of medicinal plants by *M. ruber* BCRC 31535 and its gene expression of *M. ruber* BCRC 31535-fermented *G. uralensis* and rice by RT-qPCR. The log(2) fold change of *M. ruber* BCRC 31,535-fermented *G. uralensis* to *M. ruber* BCRC 31,535-fermented rice by monacolin K gene expression was depicted on the right side of the figure. The 18 S rRNA was used as the positive control to normalize the data. Results are mean ± S.D. (n = 3)

Monacolin K was obviously detected in the fermentation of *A. pubescens*, *P. cablin*, *P. lactiflora*, *M. leucadendron*, *G. uralensis*, *P. chinense*, and rice (Fig. 6). It was not detected during the fermentation of *L. angustifolia* and *O. fragrans*. Monacolin K content in these fermentation products increased, similar to that of the red pigment during fermentation, except in *P. chinense*. The content of monacolin K derived through the 60 and 120 days fermentation of *G. uralensis* was the highest, which was 41.9-fold and 9.1-fold that in rice, respectively. Thus, the gene expression of monacolin K containing *mokA*-*mokI* genes was further analyzed by comparing *M. ruber* BCRC 31535-fermented *G. uralensis* and rice. The results indicated that the gene expression levels of monacolin K obtained through the 60-day fermentation of *G. uralensis* were higher than those in rice. However, monacolin K gene expression level after 120 days *G. uralensis* fermentation was obviously lower than that in rice. This result was consistent with monacolin K content. The content after 120 days *G. uralensis* fermentation was only 1.2 times higher than that after 60 days of fermentation. Monacolin K content in rice after 120 days of fermentation was 5.7-fold that after 60 days of fermentation.

### Total phenolic content and metabolites analysis of *M. ruber* BCRC 31535-fermented product

**Fig. 7:**
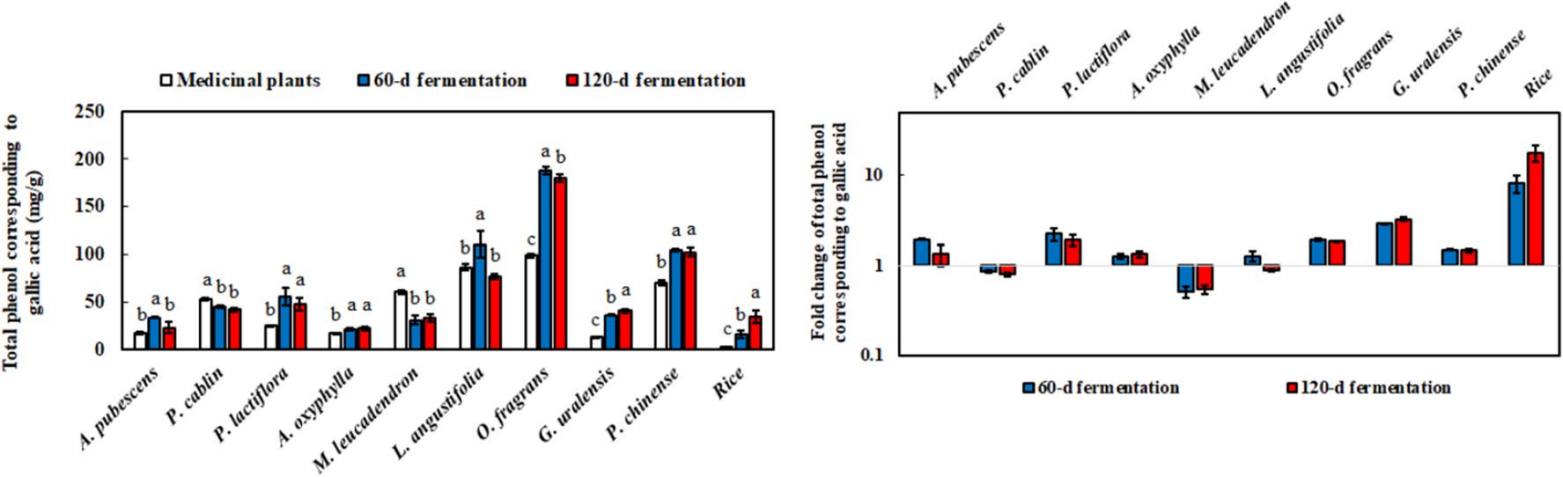
Total phenols using Folin-Ciocalteu method with or without fermentation of medicinal plants by *M. ruber* BCRC 31535. The ratio of 60 and 120 days of fermentation to medical plants was depicted on the right side of the figure. Results are mean ± S.D. (n = 3)

The antioxidant effect of the 60-day fermentation product significantly increased relative to the antioxidant effects of medicinal plants without fermentation. Accordingly, the contribution of total phenolic content to antioxidant capacity was evaluated. The result showed that the total phenolic content was elevated after *M. ruber* BCRC 31535 fermentation, except in *P. cablin*, and *M. leucadendron* (Fig. 7). The highest increase in total phenolic content was found in rice after fermentation, followed by that in *G. uralensis*. This result was consistent with the DPPH scavenging capacity.

The 60-day fermentation of *G. uralensis* enhanced DPPH scavenging, anti-inflammatory ability, monacolin K content, and total phenolic content. The metabolites of *G. uralensis* with or without fermentation were determined through LC/MS (Additional file 2: Fig. S2). The result indicated that the five compounds with the highest increases after 60 days of fermentation were 11beta-hydroxyandrost-4-ene-3,17-dione (steroid), 3,4-dihydroxyphenylglycol (phenol), leucine (amino acid), 2,3-dihydroxybenzoic acid (iron chelator), and 3-amino-4-hydroxybenzoate (monohydroxybenzoic acid), as shown in Table 2 and Additional file 3: Fig. S3. The order of relative intensity was 2,3-dihydroxybenzoic acid > leucine > 3-amino-4-hydroxybenzoate > 3,4-dihydroxyphenylglycol. The antioxidant and anti-inflammatory capacities of 2,3-dihydroxybenzoic acid against DPPH free radicals and lipopolysaccharide-induced inflammation have been demonstrated (Adjimani and Asare 2015; Alvarez Cilleros et al. 2020). Thus, 3-amino-4-hydroxybenzoate and 3,4-dihydroxyphenylglycol were further used to verify their antioxidant and anti-inflammatory abilities.

**Table 2:** The metabolites using LC/MS analysis with most increase after the 60-day *G. uralensis* fermentation by *M. ruber* BCRC 31,535

Compounds	60-day fermentation (Relative intensity)	*G. uralensis* (Relative intensity)	Fold change	Log2(fold change)
11beta-Hydroxyandrost-4-ene-3,17-dione	1.7995 ± 0.1111	0.0023 ± 0.0006	771.82	9.5921
3,4-Dihydroxyphenylglycol	13.5409 ± 0.9084	0.0465 ± 0.02575	291.41	8.1869
Leucine	30.0989 ± 1.8297	0.1727 ± 0.0992	174.3	7.4455
2,3-Dihydroxybenzoic acid	55.09045 ± 4.9859	0.382895 ± 0.2036	143.88	7.1687
3-Amino-4-hydroxybenzoate	14.7540 ± 0.8378	0.019943 ± 0.0197	89.138	6.478
Scopoletin	4.3951 ± 0.2914	0.1874 ± 0.0148	23.448	4.554
*N*-Butyryl-l-homoserine lactone	5.2381 ± 1.1549	0.2710 ± 0.0255	19.326	4.2725
Allantoic acid	2.9601 ± 0.1018	0.1624 ± 0.1627	18.229	4.1882
16-Hydroxy hexadecanoic acid	3.5948 ± 0.4486	0.2038 ± 0.0670	17.641	4.1408
Histamine	1.4013 ± 0.1212	0.0799 ± 0.0045	17.542	4.1327

### Antioxidant and anti-inflammatory analysis of 3,4-dihydroxyphenylglycol, and 3-amino-4-hydroxybenzoate

**Fig. 8:**
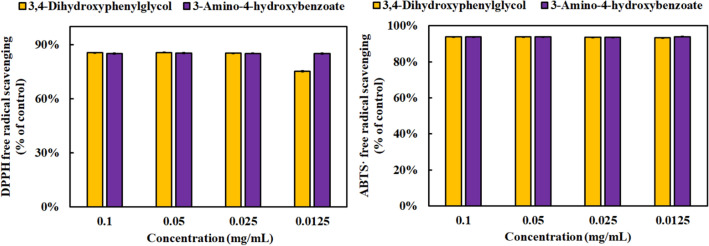
Antioxidant capacity of 3,4-dihydroxyphenylglycol and 3-amino-4-hydroxybenzoate using DPPH and ABTS scavenging methods. Results are mean ± S.D. (n = 3)

The antioxidant capacities of 3,4-dihydroxyphenylglycol and 3-amino-4-hydroxybenzoate were analyzed with DPPH and ABTS methods (Fig. 8). The result showed that over 75% DPPH scavenging activity was observed in 3,4-dihydroxyphenylglycol and 3-amino-4-hydroxybenzoate at 0.0125 mg/mL. However, 3-amino-4-hydroxybenzoate showed better antioxidation than 3,4-dihydroxyphenylglycol. The removal rate of ABTS free radical was over 90% at 0.0125 mg/mL in both compounds.

**Fig. 9:**
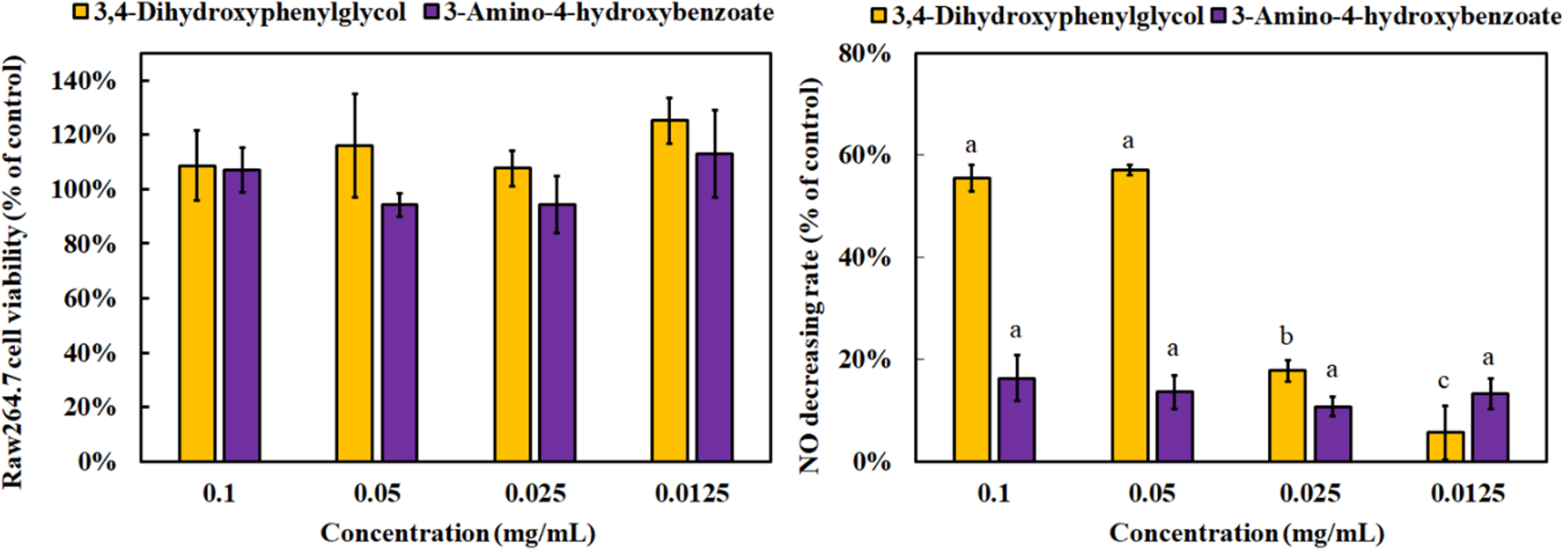
Raw264.7 cell viability and anti-inflammatory capacity of 3,4-dihydroxyphenylglycol and 3-amino-4-hydroxybenzoate using MTT method and LPS-stimulated Raw264.7 cells. Results are mean ± S.D. (n = 3)

The anti-inflammatory capacities of 3,4-dihydroxyphenylglycol and 3-amino-4-hydroxybenzoate were explored on the basis of cell viability (Fig. 9). The survival rates of Raw264.7 cells treated with 3,4-dihydroxyphenylglycol and 3-amino-4-hydroxybenzoate below 0.1 mg/mL were over 90%, and no cytotoxicity was observed. Therefore, the anti-inflammatory capacities of the two compounds were further measured. The anti-inflammatory capacity of 3,4-dihydroxyphenylglycol was observed in a dose-dependent manner. Over 56% decrease in NO was detected at 0.05 mg/mL. However, the anti-inflammatory effects of 3-amino-4-hydroxybenzoate were not significant in a dose-dependent manner.

## Discussion

*Monascus* species offer various health benefits to human beings. However, hepato-nephrotoxic citrinin of polyketide metabolites is present in *Monascus* species (de Oliveira Filho et al. 2017). Hence, many studies have focused on improving the ratio of monacolin K to citrinin through optimal cultivation or fermentation. Given that citrinin and monacolin K biosynthetic gene clusters are present in *Monascus* species (Chen et al. 2008b; Shimizu et al. 2005), considerable effort has been devoted to identifying these genes in different *Monascus* strains. According to our previous study, *pksCT*, *ctnA*, and *orf3* genes, which encode citrinin polyketide synthase, a major activator, and oxygenase, were distributed in *M. purpureus* and *Monascus kaoliang*, but not in *M. pilousus*, *M. ruber*, *M. barkeri*, *Monascus floridanus*, *Monascus lunisporas*, and *Monascus pallens* (Chen et al. 2008a). Interestingly, in this study, *mokA* and *mokE* genes were detected in *M. pilousus*, *M. ruber*, and *M. barkeri* only, and no monacolin K content was detected in *M. barkeri*. Further investigation into the citrinin and monacoin K genes of *Monascus* has shown that the distribution of citrinin and monacolin K is not restricted in the specific *Monascus* species. Some *Monascus* species simultaneously have two gene clusters, but some have only one (Chen et al. 2008a; Li et al. 2020b; Yang et al. 2015). Information about the evolutionary process of the polyketide gene cluster in fungi is currently limited, and thus gene duplication or horizontal gene transfer is worthy of further study (Wisecaver et al. 2014). On the other hand, gene sequencing similarity of monacolin K among *Monascus* species was highly homologous. This finding is in agreement with the study of Dai et al., who compared the monacolin K gene cluster of seven *M. pilosus* and *M. ruber* strains (Dai et al. 2021). The highly homologous citrinin genes in *Monascus* spp. can be also observed (Chen et al. 2008a; Li et al. 2020b). Therefore, *Monascus* species-produced monacolin K without citrinin biosynthetic genes can be screened with the complementary methods of PCR and HPLC. Consequently, *M. pilosus* BCRC 38072 and *M. ruber* BCRC 31533, 31523, 31534, 31535, and 33323 producing monacolin K without citrinin can be safely applied to the food industry.

Raw materials, such as rice and grains, provide carbohydrates, protein, and inorganic elements for traditional fermentation. Thus, considerable attention has been devoted to the fermentation of *Monascus* species with rice and grains (Jin and Pyo 2017; Maric et al. 2019; Srianta et al. 2016; Zhang et al. 2018a). Given that medicinal plants themselves have certain efficacy, fermentation through the biotransformation of microorganisms can improve the original properties of the plants and result in the production of novel chemical components (Li et al. 2020a). Scant attention has been devoted to the relationship between the fermentation of medicinal plants and *Monascus* species. Hence, several medicinal plants and rice were used in the fermentation of *M. ruber* BCRC 31535 for 60 and 120 days. Most medicinal plants and their extracts have antioxidant capacities (Chen et al. 2017; Kim et al. 2017, 2019; Soheili and Salami 2019; Surh and Yun 2012; Wu et al. 2021, 2022; Yuan et al. 2020; Zhang et al. 2018b). In this study, after the 60-day fermentation of *M. ruber* BCRC 31535 with medicinal plants, the DPPH scavenging rate significantly improved, except in *M. leucadendron* and *L. angustifolia* as raw materials. *M. leucadendron* and *L. angustifolia* contributed to antifungal activity and probably affected *M. ruber* BCRC 31535 fermentation (Abd Rashed et al. 2021; Valdes et al. 2008). Additionally, the long-term fermentation of medicinal plants (120 days) decreased antioxidant activity in the DPPH and ABTS assays. This effect may have promoted the formation of acids from antioxidant compounds, such as phenols, through long-term fermentation (Lee et al. 2019). Phenols are responsible for antioxidant activity, and the relationship between total phenolic content and antioxidant capacity was demonstrated (Additional file 4: Table S1, Additional file 5: Table S2, Additional file 6: Table S3). According to the Pearson correlation between total phenolic content and antioxidant activity, the significant effects of total phenolic content on DPPH and ABTS scavenging abilities were observed. Thus, the optimal fermentation time was important to the acquisition of antioxidants and prevention of further degradation by microbes.

Before the anti-inflammatory capacities of fermentations product generated by lipopolysaccharide-induced Raw264.7 cells were determined, cell viability was analyzed with the MTT method. Most medicinal plants were not cytotoxic. The effects of medicinal plants and fermentation products on cell viability across temporal change was evaluated by repeated-measures ANOVAs (Additional file 7: Table S4). The result indicated that cell viability was significantly affected by medicinal plants over time. This result was contrary to our expectation that toxicity would be reduced after fermentation (Li et al. 2020a). However, a survival rate of over 90% was still observed in the fermentation of *P. lactiflora*, *A. oxyphylla*, *G. uralensis*, and rice, which originally had potential anti-inflammatory activities (Okonogi et al. 2018; Xin et al. 2019; Yin et al. 2018; Zhang et al. 2018b). Thus, these medicinal plants and fermentation products were further used in the analysis of anti-inflammatory capacity. Only 60-day *G. uralensis* fermentation revealed a significant effect on NO decreasing rate. This benefit was consistent with the fermentation of ginger and tea by *M. pilosus* and *M. purpureus*, respectively (Chen et al. 2010a; Deng et al. 2021).

Red pigment and monacolin K derived from polyketides were the major characteristics in *Monascus* species. The amounts of red pigment and monacolin K increased with fermentation time in most fermentation products, and their maximum amounts were obtained after the fermentation of rice and *G. uralensis*, respectively. The repeated-measures ANOVAs demonstrated that the red pigment and monacolin K were significantly affected by medicinal plants over fermentation time (Additional file 8: Table S5). The trend was different from the trends of antioxidant and anti-inflammatory capacities. The stable production of red pigment and monacolin K was similar to the different studies using agro-industrial residues, cereal, and millet as fermented substrates (Da Silva et al. 2021; Maric et al. 2019; Srianta et al. 2016; Zhang et al. 2018a). In addition, the transcription level of monacolin K biosynthetic genes between the fermentation of *G. uralensis* and rice was further verified with RT-qPCR. As expected, the expression levels of monacolin K biosynthetic genes in the 60-day *G. uralensis* fermentation were higher than those in rice. Furthermore, no up-regulation was observed in the 120-day *G. uralensis* fermentation, and rate of increase in monacolin K content was lower than that in the 120-day rice fermentation. Moreover, MokG encoding HMG-CoA reductase may confer resistance on monacolin K (Abe et al. 2002; Chen et al. 2008b); therefore, its relative gene expression was lower than the expression levels of other genes.

LC/MS was used in analyzing difference between metabolites with or without fermentation of *M. ruber* BCRC 31533 in *G. uralensis*. The relative abundance of glutamic acid in *G. uralensis* without fermentation was 3.1-fold higher than that in 60 days fermentation. This result suggested that the consumption of glutamic acid promoted monacolin K production (Zhang et al. 2019a). In addition, *G. uralensis*, commonly known as licorice, is usually used in the food industry as a sweetener and contains various bioactive constituents, such as liquiritin, isoliquiritin, liquiritigenin, and isoliquiritigenin (Kao et al. 2014). Liquiritigenin and isoliquiritigenin are the aglycone forms of liquiritin and isoliquiritin, respectively. The relative abundance rates of liquiritin and liquiritigenin were high in *G. uralensis* and were 632.9- and 3.8-fold those in 60-day fermentation, respectively. This result suggested that the bioactive substances were metabolized and consumed by *M. ruber* BCRC 31,535. This result was different from that of Kim et al., who reported that liquiritigenin and isoliquiritigenin content improved after the fermentation of *Monascus albidulus* (Kim et al. 2020). Furthermore, 2,3-dihydroxybenzoic acid had the highest content, which increased 143.9-fold after 60-day *G. uralensis* fermentation. According to the previous study, 2,3-dihydroxybenzoic acid displayed good antioxidant and anti-inflammatory capacities (Adjimani and Asare 2015; Alvarez Cilleros et al. 2020). Moreover, 3,4-dihydroxyphenylglycol and 3-amino-4-hydroxybenzoate increased 291.4- and 89.1-fold through the biotransformation of *M. ruber* BCRC 31,535, respectively. The two compounds had high antioxidant capacities according to the DPPH and ABTS assays. The NO rate of 3,4-dihydroxyphenylglycol decreased in a dose-dependent manner. These results implied that 2,3-dihydroxybenzoic acid, 3,4-dihydroxyphenylglycol, and 3-amino-4-hydroxybenzoate improved antioxidative and anti-inflammatory effects in the 60-day fermentation of *G. uralensis*.

## Conclusions

Traditionally, the effective substance, new compound, resource conservation, and toxic reduction of medicinal plants can be enhanced by means of microbial fermentation. In this study, the antioxidant capacity and total phenolic content of medicinal plants increased after 60 days of fermentation by *M. ruber* BCRC31535. However, cytotoxicity was also elevated but not influenced in *P. lactiflora*, *A. oxyphylla*, *G. uralensis*, and rice. The positive correlation between total phenolic content and DPPH and ABTS scavenging activities was verified by repeated-measures ANOVAs. Long-term fermentation (120 days) decreased antioxidant efficacy and cytotoxicity. Red pigment and monacolin K derived from *M. ruber* BCRC31535 biosynthesis accumulated over time during fermentation. Among these medicinal plants, *M. ruber* BCRC31535-fermented *G. uralensis* had a broad spectrum of efficacy with antioxidative and anti-inflammatory capacities, and monacolin K contributed by the increase in 2,3-dihydroxybenzoic acid, 3,4-dihydroxyphenylglycol and 3-amino-4-hydroxybenzoate levels and glutamic acid consumption rates. To the best of our knowledge, this study is the first to report the antioxidative capacities of 3-amino-4-hydroxybenzoate for DPPH and ATBS scavenging activities.

## Supplementary Information

**Additional file 1: Figure S1.** Phylogeny of the partial *mokA* (a) and *mokE* (b) from *Monascus* species, and the related genes from *A. terreus*, and *P. citrinum*.

**Additional file 2: Figure S2.** LC/MS analysis with or without *M. ruber* BCRC 31535-fermented *G. uralensis* in positive and negative modes.

**Additional file 3: Figure S3.** The metabolites of the ten compounds with the most increase after 60-day *G. uralensis* fermentation in MS/MS spectrum.

**Additional file 4: Table S1.** Pearson correlation between total phenols and antioxidant activity by medicinal plants without fermentation.

**Additional file 5: Table S3.** Pearson correlation between total phenols and antioxidant activity by 120 days fermentation of *M. ruber* BCRC 31535.

**Additional file 6: Table S2.** Pearson correlation between total phenols and antioxidant activity by 60 days fermentation of *M. ruber* BCRC 31535.

**Additional file 7: Table S4.** Significance of p-value for repeated measures ANOVA on cell viability from the *M. ruber* BCRC 31535-fermentation of various medicinal plants.

**Additional file 8 Table S5.** Significance of p-value for repeated measures ANOVA on red pigment and monacolin K from the *M. ruber* BCRC 31535-fermentation of various medicinal plants and rice.

## Data Availability

The data used and analyzed in this study can be provided from the corresponding author for scientific, non-profit purpose.
