# Supplementary material for: Identification of the high-yield monacolin K strain from Monascus spp. and its submerged fermentation using different medicinal plants
Source: Bot Stud. 2022 Jul 2;63:20. doi: 10.1186/s40529-022-00351-y (PMC9250582; doi:10.1186/s40529-022-00351-y)
Supplement: Supplementary file 1 — Additional file 1: Figure S1. Phylogeny of the partial mokA (a) and mokE (b) from Monascus species, and the related genes from A. terreus, and P. citrinum. [file 40529_2022_351_MOESM1_ESM.docx]

**
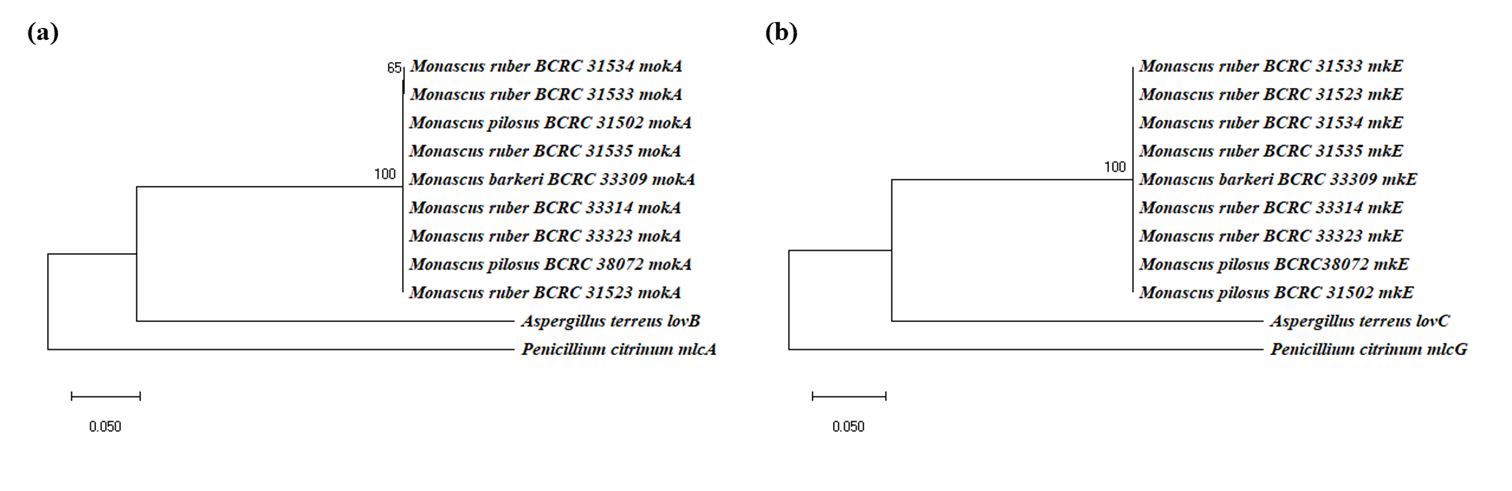
**

**Figure S1.** Phylogeny of the partial *mokA* (a) and *mokE* (b) from *Monascus* species, and the related genes from *A. terreus*, and *P. citrinum*.
