# Supplementary material for: Identification of the high-yield monacolin K strain from Monascus spp. and its submerged fermentation using different medicinal plants
Source: Bot Stud. 2022 Jul 2;63:20. doi: 10.1186/s40529-022-00351-y (PMC9250582; doi:10.1186/s40529-022-00351-y)
Supplement: Supplementary file 2 — Additional file 2: Figure S2. LC/MS analysis with or without M. ruber BCRC 31535-fermented G. uralensis in positive and negative modes. [file 40529_2022_351_MOESM2_ESM.docx]

**
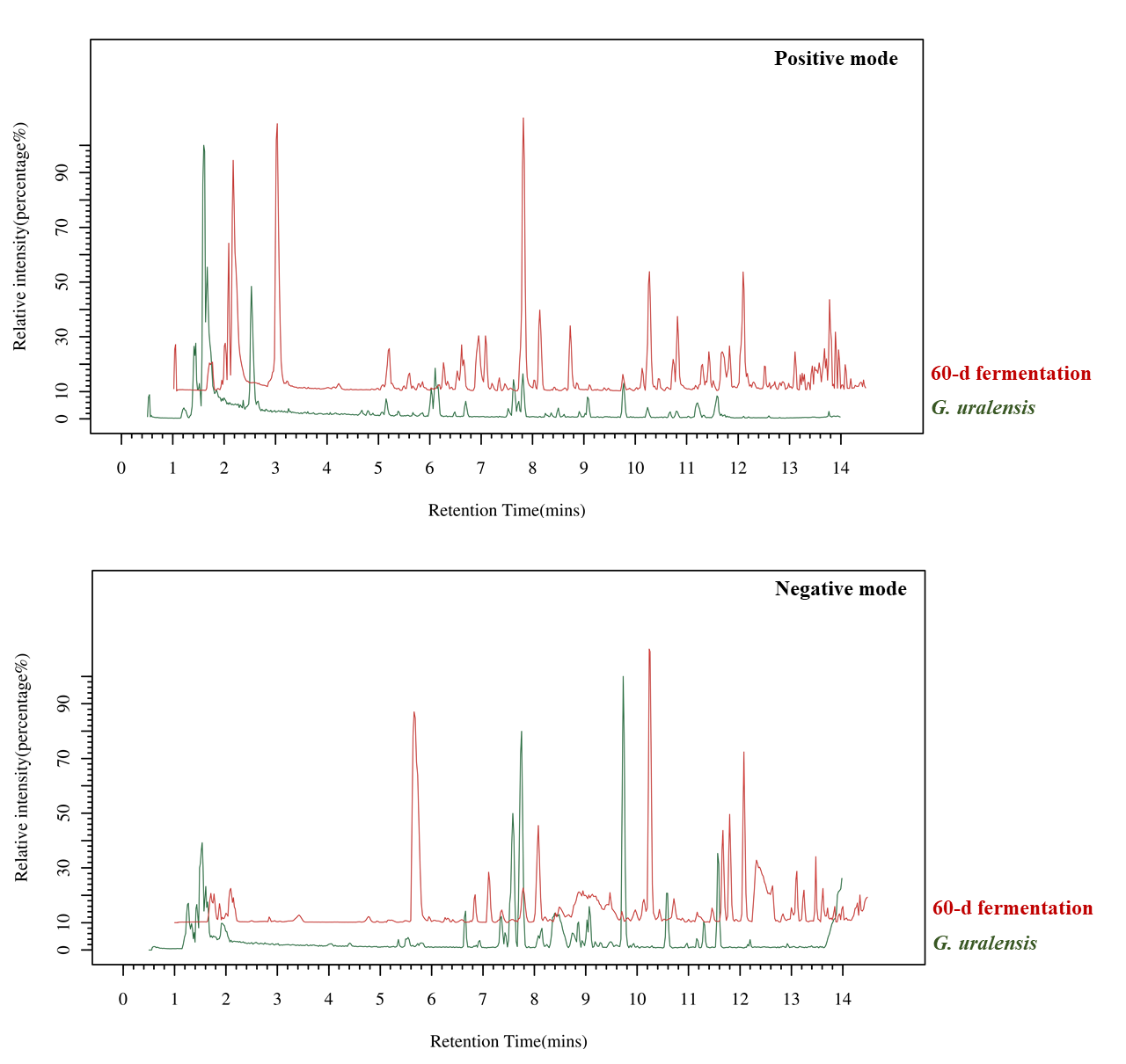
**

**Figure S2.** LC/MS analysis with or without *M. ruber* BCRC 31535-fermented *G. uralensis* in positive and negative modes.
