# Supplementary material for: Identification of the high-yield monacolin K strain from Monascus spp. and its submerged fermentation using different medicinal plants
Source: Bot Stud. 2022 Jul 2;63:20. doi: 10.1186/s40529-022-00351-y (PMC9250582; doi:10.1186/s40529-022-00351-y)
Supplement: Supplementary file 3 — Additional file 3: Figure S3. The metabolites of the ten compounds with the most increase after 60-day G. uralensis fermentation in MS/MS spectrum. [file 40529_2022_351_MOESM3_ESM.docx]

**
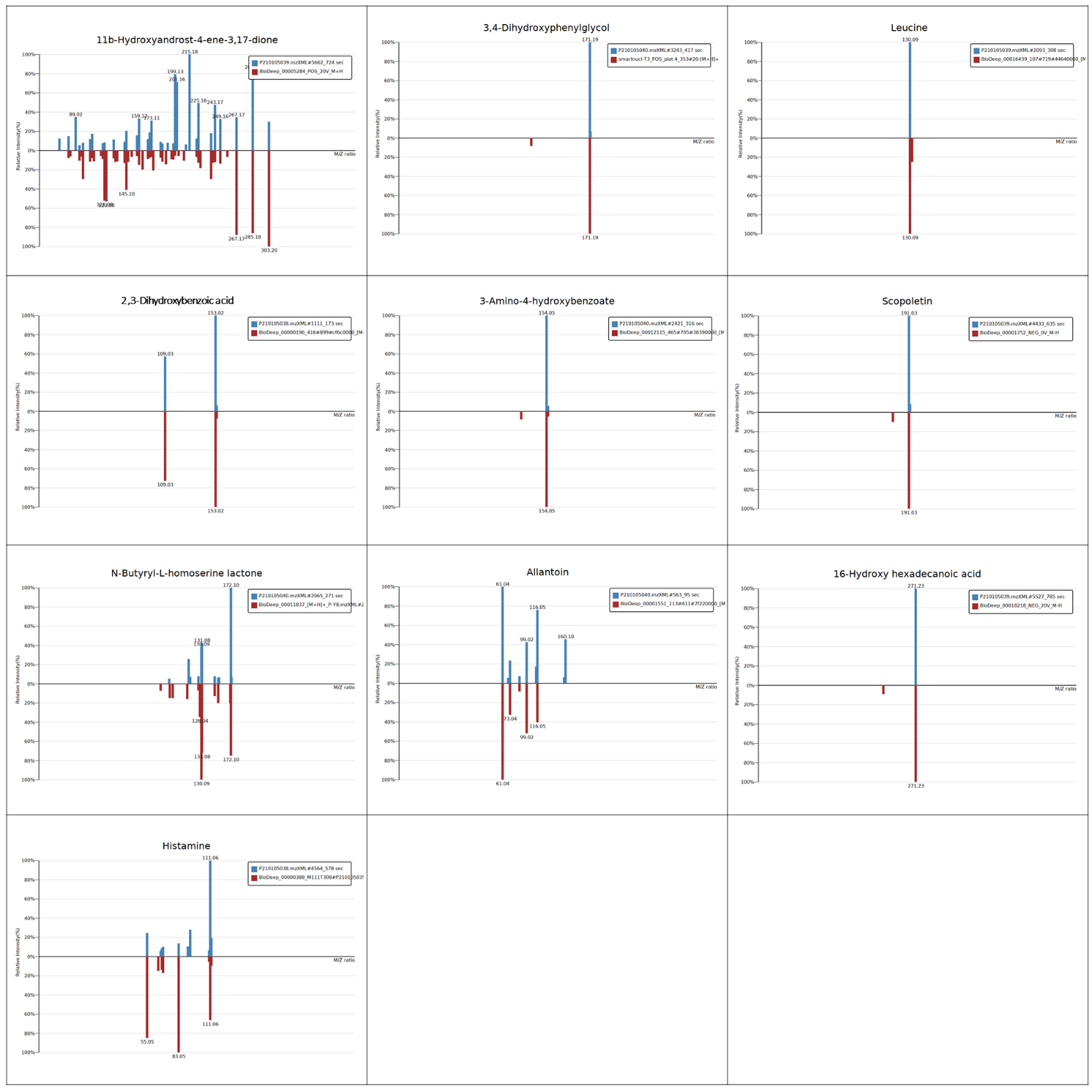
**

**Figure S3.** The metabolites of the ten compounds with the most increase after 60-day *G. uralensis* fermentation in MS/MS spectrum.
