# Supplementary material for: Identification of the high-yield monacolin K strain from Monascus spp. and its submerged fermentation using different medicinal plants
Source: Bot Stud. 2022 Jul 2;63:20. doi: 10.1186/s40529-022-00351-y (PMC9250582; doi:10.1186/s40529-022-00351-y)
Supplement: Supplementary file 4 — Additional file 4: Table S1. Pearson correlation between total phenols and antioxidant activity by medicinal plants without fermentation. [file 40529_2022_351_MOESM4_ESM.docx]

**Table S1.** Pearson correlation between total phenols and antioxidant activity by medicinal plants without fermentation.

| Medicinal plants | Total phenols | DPPH scavenging activity | ABTS scavenging activity |
| --- | --- | --- | --- |
| Total phenols |  | 0.945** | 0.843** |
| DPPH scavenging activity | 0.945** |  | 0.723** |
| ABTS scavenging activity | 0.843** | 0.723** |  |

Significance is indicated by **p-value < 0.01.
