# Supplementary material for: Identification of the high-yield monacolin K strain from Monascus spp. and its submerged fermentation using different medicinal plants
Source: Bot Stud. 2022 Jul 2;63:20. doi: 10.1186/s40529-022-00351-y (PMC9250582; doi:10.1186/s40529-022-00351-y)
Supplement: Supplementary file 5 — Additional file 5: Table S3. Pearson correlation between total phenols and antioxidant activity by 120 days fermentation of M. ruber BCRC 31535. [file 40529_2022_351_MOESM5_ESM.docx]

**Table S2.** Pearson correlation between total phenols and antioxidant activity by 60 days fermentation of *M. ruber* BCRC 31535.

| 60 days fermentation | Total phenols | DPPH scavenging activity | ABTS scavenging activity |
| --- | --- | --- | --- |
| Total phenols |  | 0.665** | 0.703** |
| DPPH scavenging activity | 0.665** |  | 0.687** |
| ABTS scavenging activity | 0.703** | 0.687** |  |

Significance is indicated by **p-value < 0.01.
