# Supplementary material for: Identification of the high-yield monacolin K strain from Monascus spp. and its submerged fermentation using different medicinal plants
Source: Bot Stud. 2022 Jul 2;63:20. doi: 10.1186/s40529-022-00351-y (PMC9250582; doi:10.1186/s40529-022-00351-y)
Supplement: Supplementary file 7 — Additional file 7: Table S4. Significance of p-value for repeated measures ANOVA on cell viability from the M. ruber BCRC 31535-fermentation of various medicinal plants. [file 40529_2022_351_MOESM7_ESM.docx]

**Table S4.** Significance of p-value for repeated measures ANOVA on cell viability from the *M. ruber* BCRC 31535-fermentation of various medicinal plants.

| Model term | Cell viability | |
| --- | --- | --- |
|  | *F* | *P* |
| Test of within-subjects effects |  |  |
| Time | 258.545 | **<0.001**** |
| Time × medicinal plants | 12.393 | **<0.001**** |
| Test of between-subjects effects |  |  |
| Intercept | 7096.569 | **<0.001**** |
| Medicinal plants | 22.07 | **<0.001**** |

Significance is indicated by **p-value < 0.01. *F* and *P* indicate the probability and significance test.
