# Supplementary material for: Identification of the high-yield monacolin K strain from Monascus spp. and its submerged fermentation using different medicinal plants
Source: Bot Stud. 2022 Jul 2;63:20. doi: 10.1186/s40529-022-00351-y (PMC9250582; doi:10.1186/s40529-022-00351-y)
Supplement: Supplementary file 8 — Additional file 8 Table S5. Significance of p-value for repeated measures ANOVA on red pigment and monacolin K from the M. ruber BCRC 31535-fermentation of various medicinal plants and rice. [file 40529_2022_351_MOESM8_ESM.docx]

**Table S5.** Significance of p-value for repeated measures ANOVA on red pigment and monacolin K from the *M. ruber* BCRC 31535-fermentation of various medicinal plants and rice.

| Model term | Red pigments | | Monacolin K | |
| --- | --- | --- | --- | --- |
|  | *F* | *P* | *F* | *P* |
| Test of within-subjects effects |  |  |  |  |
| Time | 95.252 | **<0.001**** | 24.651 | **<0.001**** |
| Time × medicinal plants | 18.763 | **<0.001**** | 8.636 | **<0.001**** |
| Test of between-subjects effects |  |  |  |  |
| Intercept | 808.264 | **<0.001**** | 268.183 | **<0.001**** |
| Medicinal plants | 11.531 | **<0.001**** | 47.545 | **<0.001**** |

Significance is indicated by **p-value < 0.01. *F* and *P* indicate the probability and significance test.
